# Clinical feasibility of two cardiac deep learning cine magnetic resonance imaging sequences: Single-breath-hold and free-breathing motion-corrected approaches

**DOI:** 10.1016/j.jocmr.2025.101983

**Published:** 2025-10-31

**Authors:** Huihui Kong, Zhaozhao Wang, Zekun Zhou, Dan YU, Guang Li, Jinchen Li, Jianmin Yuan, Xiangming Li, Yi He

**Affiliations:** a Department of Radiology, Beijing Friendship Hospital, Capital Medical University, Beijing, China; b MR Research Collaboration, Shanghai United Imaging Healthcare Co., Ltd. Shanghai, China; c Academy of Medical Engineering and Translational Medicine, Tianjin University, Tianjin, China; d R&D Center, Beijing Shuxi Technology Co., Ltd, Beijing, China

**Keywords:** Deep learning, Cine, Accelerated imaging, Free-breathing

## Abstract

**Background:**

Cine cardiovascular magnetic resonance (CMR) faces the challenges of prolonged examination times and repeated breathhold (BH). This study evaluated the clinical feasibility of deep learning (DL)-accelerated cine sequences, which shorten the acquisition time (AT) while achieving comparable image quality (IQ) and function.

**Methods:**

This prospective study included patients who underwent 3T CMR from August 2024 to March 2025. The examination included three cine sequences (2D segmented cine, 2D single-BH DL cine, and 2D free-breathing motion-corrected DL cine [FB-MOCO DL cine]). The actual total AT (including the time for short and long-axis scans, BH instructions, and resting time between BHs) was recorded. The overall IQ, blood pool to myocardium signal ratio (BMC), edge sharpness, three-dimensional volumetric mesh contour quality, biventricular cardiac function parameters, and left ventricular (LV) strain parameters were evaluated. The Friedman test was used to compare the above parameters among the three cine sequences. Correlation analysis and Bland-Altman analysis were used to evaluate the correlation and consistency between the two cine sequences.

**Results:**

Eighty-six patients were evaluated (52.98 ± 14.34 years, 79% 68/86 male). Compared with segmented cine (239.70 [224.55, 260.15])s, the total AT of single-BH DL cine (63.55 [60.98, 66.00])s and FB-MOCO DL cine (90.65 [79.43, 103.80])s decreased by 73% and 62%, respectively. There were no statistically significant differences in overall IQ and biventricular functional parameters among the three cine sequences. The three-dimensional volumetric mesh contour scores of the single-BH DL cine and FB-MOCO DL cine were higher than those of the segmented cine (P<0.001 and 0.04), but the edge sharpness and BMC were lower than those of the segmented cine. The LV strain of the two fast cine sequences was lower than that of the segmented cine.

**Conclusion:**

Compared with traditional segmented cine, DL-accelerated cine enables ventricular imaging in a shorter acquisition time, with preserved IQ and quantitative cardiac function results.

## Graphical abstract

**Figure:**
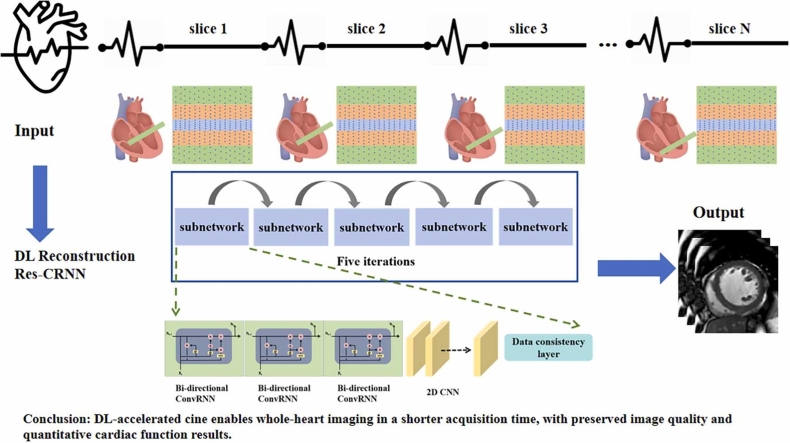

## 1 Background

Cardiovascular magnetic resonance (CMR) is a critical non-invasive modality for evaluating cardiac diseases [1], [2], [3]. Cine imaging, in particular, plays a pivotal role in quantifying ventricular volumes and function, offering essential insights for cardiovascular disease diagnosis, therapeutic efficacy assessment, and prognosis prediction [4], [5]. The conventional segmented breath-hold (BH) balanced steady-state free precession (bSSFP) cine sequence is considered the non-invasive gold standard since it delivers excellent blood to myocardium contrast and temporal resolution [6]. However, it requires multiple BHs to mitigate respiratory motion. This requirement can be challenging for many patients, as prolonged or repeated BHs are often difficult to sustain and inconsistent BH amplitudes often lead to slice misalignment. Additionally, acquiring a full short-axis stack takes approximately 6 min. Patients also need rest intervals (15 s) between BHs, further prolonging scan time [6], [7], [8]. These limitations underscore the urgent need for rapid CMR cine imaging techniques that preserve image quality while providing accurate left ventricular (LV) functional measurements.

Techniques like parallel imaging and compressed sensing (CS), have been introduced to accelerate cine acquisition, but each has drawbacks [7], [9]. Parallel imaging accelerates acquisition by undersampling k-space and using multi-channel coils spatial sensitivity for reconstruction. However, it introduces noise amplification artifacts (e.g., "noise bands") at high acceleration factors [10]. CS, on the other hand, exploits sparsity in transform domains to recover full k-space information from under-sampled data, significantly reducing scan time. Nevertheless, image quality (IQ) may be compromised, and the iterative reconstruction algorithms required for CS are computationally intensive. Consequently, the total scan time is not substantially reduced compared to conventional method [11], [12], [13].

In recent years, artificial intelligence (AI) has demonstrated potential in accelerating CMR and reducing artifacts, leading to shorter overall scanning times, faster reconstruction speeds, and preserved image quality [14], [15], [16], [17], [18], [19], [20]. Despite these encouraging results [20], [21], [22], [23], [24], [25], [26], [27], [28], most AI-based cine methods remain at the proof-of-concept stage and have yet to be fully integrated into routine clinical workflows, underscoring the need for further development and validation. In this study, two accelerated cine protocols were implemented as follows: a single-BH deep learning (DL) cine and free-breathing motion-corrected DL cine (FB-MOCO DL cine). Both approaches combine aggressive k-space undersampling with DL reconstruction to capture the entire heart in either a single breath-hold or free breathing. In the case of the FB-MOCO sequence, retrospective motion correction is applied. The aim of this study was to evaluate the IQ, biventricular volume, and functional parameters from these AI cine techniques and to compare them with segmented cine imaging to assess their clinical reliability.

## 2 Materials and methods

### 2.1 Patient population

This prospective study enrolled consecutive patients who were scheduled to undergo CMR examination at our hospital from August 2024 to February 2025. Exclusion criteria included inability to obtain informed consent, claustrophobia, and contraindications to CMR. In total, 86 consecutive patients were prospectively recruited. This study was approved by the local institutional review board (No. BFHHZS20250088), and all participants provided written informed consent for imaging.

### 2.2 CMR protocol

All CMR imaging was conducted using a 3.0 Tesla scanner (uMR870, United Imaging Healthcare, Shanghai, China) equipped with a 24-channel cardiac coils and electrocardiogram (ECG) triggering. Patients were placed in a supine position, and ECG-triggered scans were initiated. Three cine images were performed in same order for each patient: BH 2D segmented ECG-triggered bSSFP cine (segmented cine), single-BH DL cine, and FB-MOCO DL cine. The short-axis cine images covered the region from the mitral valve level to the LV apex in approximately 8–10 slices. Additionally, 2-, 3-, and 4-chamber long-axis cine images were obtained. For each cine examination, acquisition times were recorded (short-axis and long-axis acquisition time, and total acquisition time). The total acquisition time includes actual scan time, breath-hold instructions, and rest intervals. The imaging parameters are presented in Table 1.

**Table 1:** Imaging parameters for the three cine sequences.

Parameters	Segmented cine	Single-BH DL cine	FB-MOCO DL cine
ECG mode	Retrospective	Retrospective	Retrospective
TR/TE (msec)	3.32/1.57	2.93/1.36	2.93/1.36
Flip angle (º)	55	55	55
FOV (mm^2^)	320 × 360	360 × 360	360 × 360
Spatial resolution (mm^3^)	1.61 × 1.61 × 8	1.88 × 1.88 × 8	1.88 × 1.88 × 8
Image matrix	199 × 224	192 × 192	192 × 192
Temporal resolution (seconds)	53	44	44
Number of slices	9	9	9
Slice thickness (mm)	8	8	8
Bandwidth (Hz/pixel)	1000	1000	1000
Reconstructed cardiac phases	25	25	25
Number of BHs	9	1	0
Data acquisition	9 heartbeats per slice	1 heartbeat per slice	1 heartbeat per slice
VPS	16	15	15
Acceleration factor	2	12.8	14.7

*BH* breath-hold, *DL* deep learning, *ECG* electrocardiogram, *FB-MOCO* free-breathing with motion correction, *FOV* field of view, *TE* repetition time, *TR* echo time, *VPS* views per segment.

The data in the table represents the specific parameters of three cine sequences.

### 2.3 Data acquisition and image reconstruction of the DL-cine

**Fig. 1:**
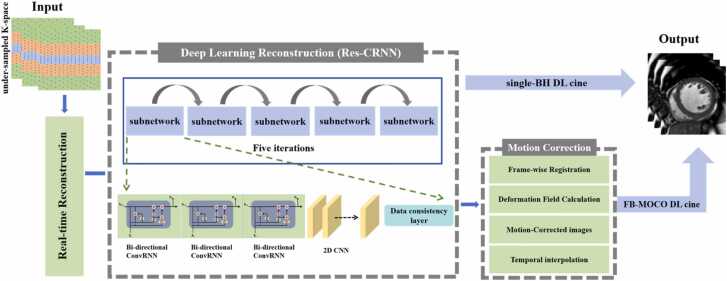
Sampling patterns and deep learning reconstruction of cine imaging. *GRU* gated recurrent unit, *CNN* convolutional neural network, *Res-CRNN* residual convolutional recurrent neural network

The standard cine sequence acquires k-space data for each slice across 9 cardiac cycles, collecting 16 k-space lines per heartbeat. Combined with parallel imaging acceleration (acceleration factor = 2), this approach balances scan efficiency and image quality. In contrast, the DL cine employs VALAS (Variable spatio-temporal LAtin hypercube and echo Sharing), which efficiently undersample the k-t space data for whole heart CMR with high spatial and temporal resolutions. The VALAS sampling scheme combines variable Latin hypercube sampling and echo-sharing with region-specific accelerations, uses echo-sharing to reduce outer acceleration, and employs sparsity-constrained reconstruction via spatial-temporal total variation optimization. Spatial-temporal total variation is implemented as an integrated regularizer within each unrolled Res-CRNN iteration: after enforcing data consistency via Nonuniform Fast Fourier Transform (NUFFT) projection, a proximal total variation (TV) step is applied in the image domain to suppress noise and enforce sparsity across space and time. Thus, echo-sharing and STTV are fully integrated into the DL reconstruction loop, not as a separate pre- or post-processing. Both DL cine protocols achieve single-heartbeat per slice acquisition (∼15 k-space lines per heartbeat) with acceleration factors of 12–15. The FB cine is based on motion correction technology, including motion tracking (monitoring respiratory and cardiac signals), deformation modeling (calculating 2D cardiac displacement/fields via frame registration), and image reconstruction (applying transformations for corrected images). Motion estimation is performed after an initial real-time reconstruction. A 2D non-rigid registration is applied frame-to-frame to estimate deformation fields representing respiratory motion [29]. All frames are deformably registered to a common respiratory reference. The estimated deformation fields are then applied to warp each original frame into this reference geometry, yielding a set of motion-corrected images that are spatially aligned across time. These motion-corrected frames, rather than pairwise-registered combinations, are used as inputs to the subsequent motion-compensated DL reconstruction. A schematic diagram summarizing the above content is provided in Fig. 1.

The DL reconstruction of the cine sequence in this study is based on a Residual Convolutional Recurrent Neural Network (Res-CRNN). By integrating spatiotemporal features through a deep neural network, it significantly enhances efficiency while ensuring accuracy, addressing the issues of time-consuming iterative optimization and limited generalization ability in traditional CS reconstruction. The Res-CRNN adopts an iterative design, comprising five iterations of non-parametrically shared sub-networks, with each iteration refining the output of the previous one. Its key components include the following: 1) A bidirectional convolutional RNN layer to capture the temporal dependencies in both forward and backward directions of the dynamic sequence. 2) 2D convolutional layers to extract spatial features, which consume less GPU memory compared to 3D convolutional layers. 3) A data consistency (DC) layer enforces physical constraints by fusing originally sampled k-space data with network predictions to ensure alignment of the reconstructed k-space. This mechanism preserves acquired data at sampled positions while utilizing network predictions elsewhere, ensuring fidelity. When embedded in architectures like Res-CRNN, the DC layer physically constrains high-frequency details learned through residual connections, suppresses artifacts, and enables end-to-end training via gradient backpropagation, thereby reducing required iterations. 4) Residual connection layers to enhance the reconstruction of high-frequency details. Furthermore, consistent with the dynamic sequence interpolation in CS reconstruction, the algorithm first reconstructs images (For single-BH cine one image slice was reconstructed for each heartbeat, and for FB cine, a sequence of real-time images) and then interpolates them to a complete cardiac cycle based on ECG time information. A dedicated reconstruction workstation ensures efficient computation. Training used retrospectively undersampled breath-hold 2D cine datasets. Ground-truth references were fully sampled multi–breath-hold 2D cine images reconstructed with the vendor’s standard pipeline (parallel imaging and routine regularization). VALAS undersampling was retrospectively applied to generate inputs, and supervised learning minimized reconstruction error to the fully sampled references. No motion correction or temporal interpolation was applied to the training data (breath-held scans do not contain respiratory motion); these steps are used only at inference for free-breathing data. Beyond the network-embedded spatio-temporal modeling (including ST-TV within unrolled iterations), no additional explicit temporal regularization was imposed during training. The trained algorithm mentioned above is provided by the supplier (Shanghai United Imaging Healthcare) and is directly installed on the magnetic resonance scanner. As this study primarily focuses on evaluating the diagnostic efficacy of DL cine in clinical applications, detailed descriptions of the network training module are deliberately omitted. Interested readers may refer to relevant literature [30], [31].

## 3 Image analysis

### 3.1 Image quality evaluation

All image analyses were performed by a radiologist with 5 years of CMR diagnostic experience. Image quality was to comprehensively assessed using overall IQ scores, blood pool myocardial signal ratio (BMC) and edge sharpness.

The short-axis cine images were scored for overall IQ using a 5-point Likert scale: 1, non-diagnostic, no clinical significance; 2, poor, obvious motion-related artifacts or image distortion, but partial diagnostic significance; 3, moderate motion-related artifacts or image distortion, but fully identify ventricular contour; 4, good, mild motion-related artifacts or image distortion; 5, excellent, no or minor motion-related artifacts or image distortion. Datasets with a score≥3 were included in the study [28], [32]. Traditional signal-to-noise ratio cannot be measured due to continuous noise removal by the reconstruction process. Therefore, quantitative metrics included the BMC, calculated by measuring the signal intensity (SI) of the LV blood pool (BP) and the septal myocardium in diastole [33].

Finally, the edge sharpness at the endocardial blood pool boundary, end-diastolic phase 4-chamber view images were imported into a custom MATLAB (version R2022a; The MathWorks, Natick, Massachusetts, USA) script for analysis. A line was manually drawn across the mid-ventricular septum. The edge sharpness was defined as the reciprocal of the distance (pixel^−1^) between two points corresponding to 20% and 80% of the maximum signal intensity difference along the line segment [7], [34].

### 3.2 LV basic cardiac function parameters

LV basic cardiac function parameters were measured by a radiologist (5 years of experience in cardiac imaging), who was blinded to patient information and clinical data. The radiologist use post-processing software Medis Suite MR (version 3.1.16.24; QMass module, Medis Medical Imaging, Leiden, The Netherlands) to process the cardiac function parameters. The epicardial and endocardial borders of short-axis cine images were automatically tracked during end-diastole and end-systole, manually adjusted if necessary. The ventricular cavities contained the endocardial trabecular and papillary muscles. These biventricular functional parameter included ejection fraction (EF), end diastolic volume (EDV), end systolic volume (ESV), cardiac output (CO), stroke volume (SV), and LV end-diastolic mass (LVmass). LV strain parameters were measured, including global circumferential strain (GCS), global longitudinal strain (GLS), global radial strain (GRS). Record three-dimensional volumetric mesh contours. The strain analysis was performed using Medis Suite MR (version 3.1.16.24; QStrain module, Medis Medical Imaging, Leiden, The Netherlands). Manual contouring was performed on the end-diastolic frames of both long- and short-axis cine images to determine the LV endocardial and epicardial borders. Then, the contours were automatically propagated to all phases and modified manually, if necessary. Papillary muscles were excluded from the endocardial contour. The cine long-axis (2-, 3-, and 4-chamber) images were used for the LV GLS analysis, and cine short-axis images (basal, mid, and apical) were used for the LV GCS and GRS analysis. We scored the image quality of the LV three-dimensional volumetric mesh contour by referring to the five-point scoring scale of this reference [28].

To evaluate agreement in LVEF, two key analyses were performed in the following: (1) determination of concordance rates between single-BH/FB-MOCO DL cine and reference-standard classifications of LV functional impairment (categorized as: normal [LVEF ≥50%], mildly impaired [45%–49%], moderately impaired [35%–44%], and severely impaired [<35%]); and (2) quantification of discordant classifications where single-BH/FB-MOCO DL cine-based LVEF estimates were misclassified into a lower or higher severity category compared to the reference standard (segmented cine).

### 3.3 Statistical analysis

All data were analyzed using SPSS software (version 25.0, IBM Corporation, Armonk, New York, USA). First, the Shapiro-Wilk test was applied to assess normality. Normally distributed continuous variables were expressed as mean ± standard deviation (SD), while non-normally distributed variables were reported as median (interquartile range). For comparisons of imaging time, overall image quality, edge sharpness, BMC, and cardiac functional parameters across the three cine sequences: One-way ANOVA was used if data met assumptions of normality and homogeneity of variance. Friedman test (nonparametric alternative for related samples) was applied for non-normally distributed data. If a significant difference was detected (P<0.05), post hoc pairwise comparisons with Bonferroni correction were performed [21]. For functional parameters with non-normal distributions showing significant differences (P<0.05), Cliff’s delta (Δ) was calculated to quantify effect size, categorized as: very small: |Δ| < 0.147, small: 0.147 ≤ |Δ| < 0.33, fair: 0.33 ≤ |Δ| < 0.474, high: |Δ| ≥ 0.474 [20]. Bland-Altman analysis was conducted to evaluate agreement between cine sequences, reporting the mean difference and 95% limits of agreement (LoA). Correlation analysis between cardiac functional parameters from two cine sequences was assessed using: Pearson’s correlation coefficient for normally distributed data; Spearman’s rank correlation coefficient for non-normally distributed data. Based on the correlation coefficient (r), it can be classified into no (r < 0.3), weak (0.3≤r<0.5), moderate (0.5≤r<0.7), and strong (r≥0.7) correlations. P <0.05 was considered to be statistically significant.

## 4 Results

### 4.1 Basic characteristics of patients

Table 2 summarizes the patient clinical characteristics. There were 86 patients (68 males; 52.98 ± 14.34 years; heart rate 67.06 ± 8.77 beats/min) who underwent all three cine scans during the study period. All cine images were obtained without technical failure or significant artifact (IQ≥2). The clinical indications of patients include myocardial infarction (n = 36), cardiomyopathy (n = 14), hypertensive heart disease (n = 6), myocarditis (n = 3), left ventricular/atrial enlargement (n = 5), heart failure (n = 8), valvular heart disease (n = 5), pericardial effusion (n = 2), negative findings (n = 6) and left atrial myxoma (n = 1).

**Table 2:** Clinical characteristics of the patients (n = 86).

Clinical characteristic	Value
Number of patients	86
Age (years)	52.98±14.34
Male (%)	68 (79%)
Female (%)	18 (21%)
Heart Rate (bpm)	67.06±8.77
*Clinical indications*	
Negative findings	6 (6.9%)
Cardiomyopathy	14 (16.2%)
Myocardial infarction	36 (41.8%)
Left ventricular/atrial enlargement	5 (5.8%)
Hypertensive heart disease	6 (6.9%)
Valvular heart disease	5 (5.8%)
Pericardial effusion	2 (2.3%)
Heart failure	8 (9.3%)
Left atrial myxoma	1 (1.1%)
Myocarditis	3 (3.4%)

Data are presented as mean±standard deviation or No. (%).

### 4.2 Comparison in acquisition time

**Fig. 2:**
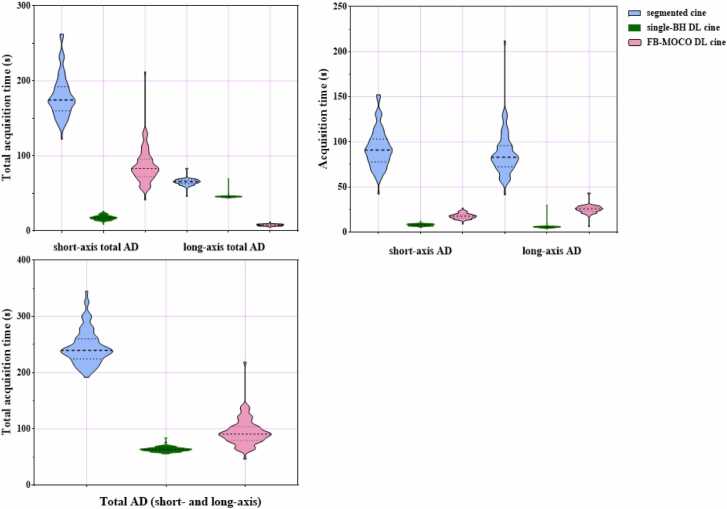
Comparison of the three cine acquisition times.

A total of 9.7 ± 0.9 slices were acquired per patient. Compared with segmented cine [short-axis: 90.95 (77.75, 103.13) s, long-axis: 25.50 (23.03, 27.73) s], the short-axis acquisition time of single-BH DL cine [17.76 ± 3.14 s, P<0.001] and FB-MOCO DL cine [83.00 (72.45, 95.78) s, P<0.001] were significantly shortened by 80% and 9%, respectively, and that of long-axis cine imaging was also shortened by 78% [5.65 (5.10, 6.13) s, P<0.001] and 69% [7.97 (6.90, 9.00) s, P<0.001] respectively. For the total acquisition time (including BH instructions and rest time between BHs), the single-BH DL cine (P<0.001) and FB-MOCO DL cine (P<0.001) were also significantly shorter compared with segmented cine. For short-axis cine imaging, the acquisition time was shortened by 90% and 52% respectively, and for long-axis cine imaging, it was shortened by 30% and 91% respectively. Compared with segmented cine (239.70 [224.55, 260.15]), the total acquisition time (short and long axis) of single-BH DL cine (63.55 [60.98, 66.00]) and FB-MOCO DL cine (90.65 [79.43, 103.80]) decreased by 73% and 62% respectively. Fig. 2 depicts the comparison of the three cine acquisition times.

### 4.3 Comparison in overall image quality

**Fig. 3:**
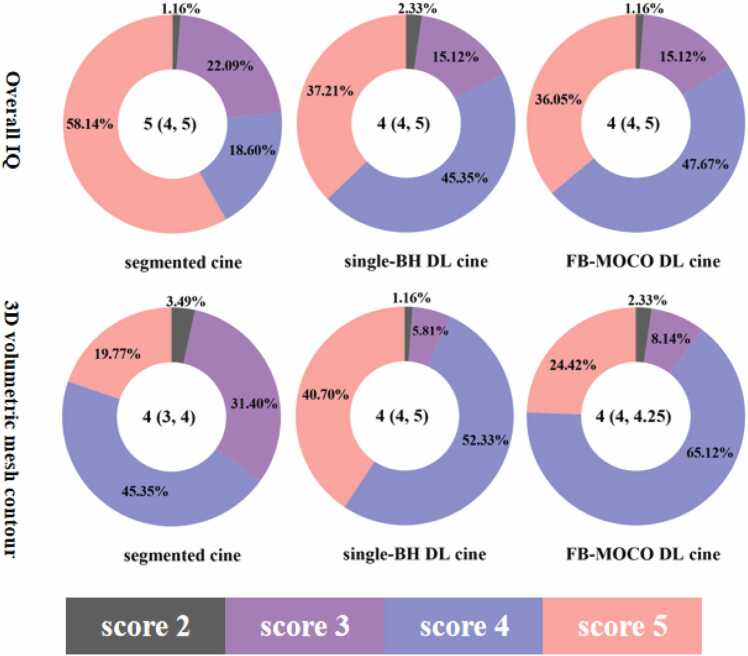
Overall IQ and three-dimensional volumetric mesh contour scores. Median (interquartile range) is indicated in the white circles, while percentages of patients scored from 1–5 are shown in the pie charts. *DL* deep learning, *BH* breath-hold, *FB* free breathing, *MOCO* motion correction, *IQ* image quality

There was no statistical difference in overall image quality between single-BH DL cine (4 [4, 5]), FB-MOCO DL cine (4 [4, 5]) and segmented cine (5[4, 5]; χ²=7.74, P=0.309). Except for three patients with IQ scores of 2 (one on segmented cine, one on single-BH DL cine, and one on both single-BH DL cine and FB-MOCO DL cine), the overall IQ scores of the remaining patients were ≥3, indicating that all three cine sequences met clinically diagnostic IQ standards across all patients. The percentage of different scores were: 5 points (58.1%, 37.2%, 36.0%, respectively for segmented-, single-BH, and FB-MOCO DL cine), 4 points (18.6%, 45.3%, 47.6%), 3 points (22%, 15.1%, 15.1%), 2 points (1.1%, 2.3%, 1.1%). Fig. 3 shows the distribution of image quality scores for the three types of cine sequences.

The average three-dimensional volumetric mesh contour scores for segmented, single-BH, and FB-MOCO DL cine were 4 (3, 4), 4 (4, 5), 4 (4, 4.25), respectively, with statistical differences (χ²=35.42, P <0.001). The pairwise comparison results showed that there were significant differences between segmented- and single-BH DL cine (Z = −4.23, P<0.001), and between segmented- and FB-MOCO DL cine (Z = −2.40, P = 0.049). However, there was no statistically significant difference between single-BH DL cine and FB-MOCO DL cine (Z=1.83, P = 0.20).

The edge sharpness for segmented, single-BH, and FB-MOCO DL cine were 0.67 (0.53, 0.77), 0.42 (0.30, 0.59), 0.45 (0.32,0.63), respectively, with statistical differences (χ²=59.21, P<0.001). The pairwise comparison results showed that there were significant differences between segmented- and single-BH DL cine (Z = 7.47, P<0.001), and between segmented- and FB-MOCO DL cine (Z = 5.11, P<0.001). The three cine sequences demonstrated consistent results with edge sharpness in BMC with statistical differences. Segmented cine demonstrated significantly higher BMC than both single-BH cine (Z = 4.12, P<0.0001) and FB-MOCO DL cine (Z = 2.52, P = 0.04). For specific numerical results, refer to Table 3.

**Table 3:** Comparison of IQ, edge sharpness, BMC, and 3D volumetric mesh contour for the three cine sequences.

Sequence	Segmented cine	Single-BH DL cine	FB-MOCO DL cine	χ² (degrees of freedom)	P
IQ	5 (4, 5)	4 (4, 5)	4 (4, 5)	7.74 (2)	0.309
Edge sharpness	0.67 (0.53, 0.77)	0.42 (0.30, 0.59)	0.45 (0.32, 0.63)	59.21 (2)	＜0.001
BMC	2.52 (2.16, 2.95)	2.38 (2.04, 2.64)	2.40 (2.08, 2.76)	17.23 (2)	＜0.001
3D volumetric mesh contour	4 (3, 4)	4 (4, 5)	4 (4, 4.25)	35.42 (2)	＜0.001

Normally distributed continuous variables were expressed as mean ± SD, while non-normally distributed variables were reported as median (interquartile range).

*DL* deep learning, *BH* breath-hold, *FB* free breathing, *MOCO* motion correction, *IQ* image quality, *BMC* blood pool myocardial signal ratio.

### 4.4 Comparison in biventricular cardiac function parameters

**Fig. 4:**
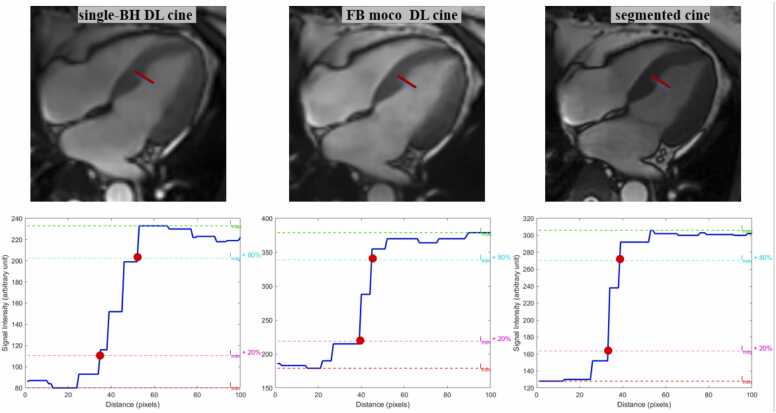
Edge sharpness at the endocardial blood pool boundary. End-diastolic 4-chamber image analysis was selected and a line was manually drawn across the mid-ventricular septum. The edge sharpness was defined as the reciprocal of the distance (pixel^−1^) between two points corresponding to 20% and 80% of the maximum signal intensity difference along the line segment. *DL* deep learning, *BH* breath-hold, *FB* free breathing, *MOCO* motion correction

Comparison of the biventricular cardiac function parameters is shown in Table 4. Among the three cine sequences, no statistically significant differences were observed in all measured biventricular volumetric, functional parameters, and LV mass (all P>0.05). Additionally, in the correlation analysis, all measured parameters demonstrated strong correlations (all r ≥ 0.80) between the single-BH/FB-MOCO DL cines and the segmented cine (Table 5, Fig. 4). Bland-Altman analysis further revealed small mean differences and narrow LoA for all CMR cardiac function parameters between the single-BH/FB-MOCO DL cines and the segmented cine, indicating excellent agreement (Table 5, Fig. 4).

**Table 4:** Comparison of LV and RV function parameters between the three cine sequences.

Parameters	Segmented cine	Single-BH DL cine	FB-MOCO DL cine	χ² (degrees of freedom)	P
LV EDV	143.65 (123.94, 170.99)	141.68 (124.06, 163.50)	144.90 (124.07, 172.34)	5.46 (2)	0.07
LV ESV	64.88 (55.92, 86.03)	65.75 (57.95, 85.48)	67.28 (57.60, 85.69)	0.13 (2)	0.94
LV SV	73.81±17.30	73.47±15.57	73.97±16.68	0.22 (2)	0.81
LV EF	53.39 (43.50, 57.94)	52.40 (44.39, 57.36)	52.93 (44.05, 57.83)	2.26 (2)	0.32
LV CO	4.83 (4.29, 5.48)	4.75(3.99, 5.49)	4.80 (4.10, 5.55)	5.93 (2)	0.05
LV MASS	104.42 (88.84, 126.31)	100.11 (84.95, 121.56)	99.91 (86.84, 118.21)	5.28 (2)	0.07
RV EDV	121.63±28.60	120.03±29.02	118.56±30.90	0.40 (2)	0.67
RV ESV	61.43±18.81	59.65±18.72	59.13±19.52	0.35 (2)	0.71
RV SV	60.20±18.55	59.02±17.68	58.19±19.71	0.15 (2)	0.86
RV EF	49.89 (44.27, 56.42)	49.96 (45.09, 56.95)	50.24 (44.84, 56.70)	5.40 (2)	0.07
LV GCS	−18.49 (−20.77, −15.05)	−15.78 ±4.57	−15.95 (−18.30, −12.93)	49.07 (2)	<0.001
LV GRS	53.61 ±19.64	42.37 (32.52, 60.12)	44.16 (34.19, 59.78)	9.85 (2)	0.007
LV GLS	−20.08 (−22.15, −16.21)	−17.00±4.54	−16.84±4.67	43.16 (2)	<0.001

Normally distributed continuous variables were expressed as mean ± SD, while non-normally distributed variables were reported as median (interquartile range).

*DL* deep learning, *BH* breath-hold, *FB* free breathing, *MOCO* motion correction, *LV* left ventricular, *RV* right ventricular, *CO* cardiac output, *EDV* end diastolic volume, *EF* ejection fraction, *ESV* end systolic volume, *SV* stroke volume, *GCS* global circumferential strain, *GLS* global longitudinal strain, *GRS* global radial strain.

**Table 5:** Results of the Bland-Altman agreement analysis and correlation analysis between the two fast cines and the segmented cine.

Parameters	Segmented cine vs. Single-BH DL cine				Segmented cine vs. FB-MOCO DL cine		
	Mean difference	95% LoA	r		Mean difference	95% LoA	r
LV EDV	0.94	(−20.39, 22.26)	0.93		−1.60	(−25.95, 22.74)	0.91
LV ESV	0.60	(−11.81, 13.01)	0.96		−1.44	(−17.30, 14.41)	0.96
LV SV	0.34	(−14.58, 15.25)	0.90		−0.16	(−12.54, 12.22)	0.93
LV EF	−0.16	(−7.28, 6.95)	0.97		0.33	(−3.93, 4.60)	0.97
LV CO	0.08	(−1.00, 1.16)	0.84		−0.02	(−1.36, 1.32)	0.82
LV MASS	3.92	(−13.32, 21.15)	0.95		3.56	(−19.01, 26.18)	0.93
RV EDV	2.05	(−20.71, 24.82)	0.92		3.85	(−20.55, 28.26)	0.91
RV ESV	1.64	(−10.66, 13.95)	0.94		2.30	(−12.34, 16.94)	0.93
RV SV	0.41	(−13.06, 13.88)	0.93		1.55	(−12.47, 15.58)	0.93
RV EF	−0.63	(−6.12, 4.86)	0.97		−0.27	(−7.04, 6.50)	0.98
LV GCS	−1.82	(−7.54, 3.91)	0.79		−1.68	(−9.23, 5.88)	0.81
LV GRS	7.73	(−29.33, 44.79)	0.54		5.93	(−31.13, 42.99)	0.49
LV GLS	−2.25	(−8.03, 3.54)	0.80		−2.40	(−9.10, 4.30)	0.72

*DL* deep learning, *BH* breath-hold, *FB* free breathing, *MOCO* motion correction, *LV* left ventricular, *RV* right ventricular, *CO* cardiac output, *EDV* end diastolic volume, *EF* ejection fraction, *ESV* end systolic volume, *SV* stroke volume, *GCS* global circumferential strain, *GLS* global longitudinal strain, *GRS* global radial strain.

The data in the table represent the mean difference values, 95% LoA values, and correlation coefficient values, respectively.

**Fig. 5:**
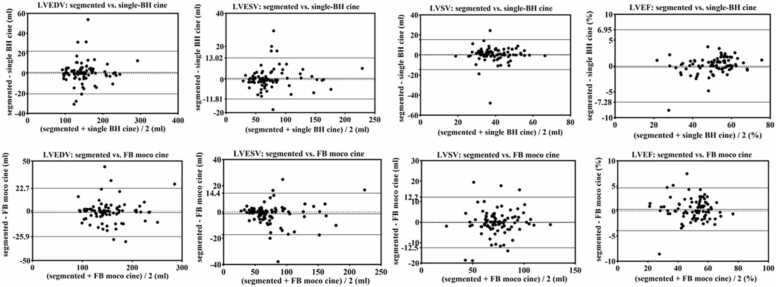
Bland-Altman plots of LVEDV, LVESV, LVSV, and LVEF showing agreement between segmented-, single-BH DL cine, and FB-MOCO DL cine. Middle black solid line represents the average difference, and black solid lines on both sides indicate 95% confidence intervals. *DL* deep learning, *BH* breath-hold, *FB* free breathing, *MOCO* motion correction, *LVEDV* enddiastolic volume of left ventricle, *LVESV* end-systolic volume of left ventricle, *LVEF* ejection fraction of left ventricle, *LVSV* stroke volume of left ventricle

**Fig. 6:**
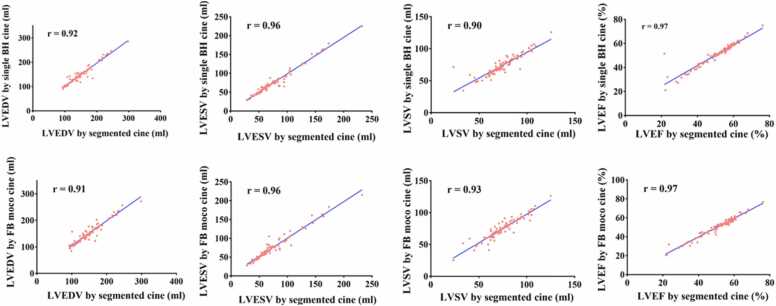
Correlation analysis comparing LVEDV, LVESV, LVSV, and LVEF between segmented cine and two DL cines. *DL* deep learning, *BH* breath-hold, *FB* free breathing, *MOCO* motion correction, *LVEDV* enddiastolic volume of left ventricle, *LVESV* end-systolic volume of left ventricle, *LVEF* ejection fraction of left ventricle, *LVSV* stroke volume of left ventricle

With regard to grading of LV EF impairment in the 86 patients, as determined with the standard of reference sequence, EF was normal in 53 patients, slightly impaired in 11, moderately impaired in 13, and severely impaired in nine. With single-BH DL cine and FB-MOCO DL cine, six and five of the 86 patients, respectively, would have been misclassified compared with the standard of reference classification.

### 4.5 Comparison in LV strain parameters

Comparison of the LV strain parameters are shown in Table 4. There were significant differences in LV strain between segmented- and single-BH DL cine (GCS: −1.82%, GRS: 7.73%, GLS, −2.25%) and between segmented- and FB-MOCO DL cine (GCS: −1.68%, GCS: 5.93%, GLS, −2.40%) (Table 5). Further effect size analysis as calculated by Cliff’s delta (Δ) showed very small difference in GRS, GCS, and GLS between segmented- and single-BH DL cine (ΔGRS = 0.26, ΔGCS = −0.24, ΔGLS = 0.30) and segmented- and FB-MOCO DL cine (ΔGRS = −0.22, ΔGCS = −0.27, ΔGLS = −0.30), indicating that the difference can be ignored. There is a strong correlation between GLS and GCS in single-BH/FB-MOCO DL cines and segmented cine (all r ≥ 0.72), with GRS showing moderate correlation (r = 0.54, 0.49, respectively).

## 5 Discussion

This prospective study validated the feasibility of two DL-based cine sequences in all patients undergoing CMR examinations for suspected heart diseases. It was found that the single–BH DL cine and the FB-MOCO DL cine sequences substantially shorten acquisition time, with the total imaging time reduced by 73% and 62%, respectively. Both DL cines achieved clinically acceptable overall IQ scores (median 4 of 5) and yielded left and right ventricular volumes, ejection fractions, and myocardial mass measurements strongly concordant with those obtained by the conventional segmented bSSFP cine, despite a modest decrease in edge sharpness that did not hinder contour delineation for volumetric analysis.

Although the standard segmented BH cine sequence remains the clinical reference standard, its dependence on multiple BHs can be problematic for patients with cardiovascular disease. Techniques that minimize or even eliminate BH requirements would therefore be highly advantageous and are primed for wider clinical adoption [14], [15], [16], [17], [18]. However, these traditional acceleration techniques (parallel imaging’s reliance on coil sensitivity and compressed sensing’s dependence on sparsity and iterative reconstruction) either offer only modest speedups or suffer from noise amplification at higher acceleration factors. In recent years, AI applications in cardiovascular imaging have demonstrated promise by speeding acquisitions, automating reconstruction, and enhancing IQ [14], yet most remain at the proof-of-concept stage.

Two broad AI-based acceleration strategies have emerged. The first acceleration technology is based on DL to realize super-resolution reconstruction. After collecting low-resolution images, high-resolution images are obtained through post-processing, such as the generative adversarial inline network by Siyeop et al. which converts undersampled low-resolution data into high-fidelity images have demonstrated significant reductions in breath-holds and scan time while maintaining functional accuracy [27]. In contrast, another accelerated technique combining k-space undersampling (CS and variable-density sampling)[21], [22], [23], [24], [25], [28] with DL reconstruction (DL-eigenvalue iterative self-consistent parallel imaging reconstruction, DL-ESPIRiT)[25], [28], [35] appear more readily translatable to clinical practice at present. This study employs a hybrid strategy that combines VALAS k-space sampling with an unrolled Res-CRNN. This design enforces strict data consistency at each iteration while the network’s learned regularization suppresses aliasing artifacts, enabling robust 12–15-fold undersampling (compared to the typical 2-fold undersampling in traditional cine imaging) without compromising image quality. By directly employing the original k-space data and coil sensitivity information into a DL model, we achieve dramatic acquisition speedups alongside robust image quality and reliable ventricular measurements. Compared to the previously widely adopted DL-ESPIRiT network [25], [28], [35], the Res-CRNN framework used in this study leverages bidirectional convRNN layers to model dynamic information. The integration of temporal and spatial convolutions enables better capture of temporal dependencies and more accurate reflection of physiological motion changes. The proposed method leverages information from adjacent frames to enhance temporal consistency and mitigate artifacts. In contrast, methods relying on separate temporal and spatial convolutions (DL-ESPIRiT network) may struggle to maintain coherence when encountering rapid motion or abrupt intensity changes between frames, as their motion modeling operates on shorter temporal contexts. Meanwhile, the Res-CRNN framework not only applies residual connections at the iteration level but also introduces two-level residual connections within the convRNN architecture. This design promotes high-frequency detail learning while precisely separating noise from signals, effectively reducing black-band artifacts and enhancing tissue contrast. These innovations demonstrate significant advantages in dynamic information modeling and high-frequency detail processing.

Additionally, it includes other DL-based reconstruction networks. Xu et al. [36] developed A-LIKNet, a multi-domain fusion network for dynamic MRI reconstruction. It uses parallel branches to independently process k-space data (preserving frequency details, reducing distortion) and image-domain data (optimizing spatial features), coupled for information exchange. An attention mechanism dynamically weights crucial coils (e.g., near the heart) and key cardiac motion phases (e.g., systole/diastole), while low-rank priors constrain spatiotemporal correlations, enabling single-BH 2D cine imaging. Qin et al. [37] proposed a DL-based method for fast dynamic multi-coil MRI reconstruction. Their core innovation is a complementary network jointly capturing correlations in the spatiotemporal-frequency (x-f) domain and spatiotemporal-image (x-t) domain. They model reconstruction as a multi-variable minimization problem, solved iteratively via variable splitting for x-f/x-t signal de-aliasing, data consistency optimization, and weighted coupling. This iterative framework is embedded in an RNN to learn spatiotemporal redundancies, offering another option for single-BH 2D cardiac cine imaging. Morales et al. [38] introduced DENT, a transformer-based neural network for cardiac motion interpolation. It uses bidirectional optical flow to precisely capture deformation features between cardiac phases. Operating in a four-frame input, single-frame output sliding window paradigm, DENT effectively doubles the temporal resolution (frame rate) of cardiac imaging sequences while preserving hemodynamic information integrity. Additionally, Küstner et al. [39] presented 4D CINENet, a novel DL network for reconstructing prospectively undersampled 3D Cartesian CINE data into 4D (3D+time). Based on (3+1)D complex-valued spatiotemporal convolutions and multi-coil processing, it achieves isotropic resolution 3D CINE imaging within a single BH, requiring under 10 s of scan time and approximately 5 s for reconstruction.

Previous studies combining K-space undersampling and DL reconstruction acceleration techniques have reported small but statistically significant discrepancies in ventricular measurements. In the study of Zucker et al. [28] on 50 children and young adults achieving FB cine imaging, compared with traditional techniques, LVSV, LVMASS, RVEDV, and RVSV had significant differences. And the image quality of DL cine was reduced. Another study of 29 children also noted differences in LVMASS and RVEDV, with slightly lower IQ [23]. Monteuuis et al. [25] and Yan et al. [20] found differences in LVEDV and LVMASS in 26 ischemic heart disease patients and 70 patients, respectively. These inconsistencies likely stem from two factors as follows: respiratory motion causing misalignment of basal and apical slices during endocardial segmentation, and blurring artifacts from aggressive undersampling that obscure myocardial borders [25]. Moreover, the small sample sizes in these pilot studies may have amplified these effects. In contrast, our study included a relatively large sample size, and the results demonstrated no significant differences in biventricular function between single-BH/FB-MOCO DL cine and segmented cine. Whereas prior DL cine studies have been limited to pediatric cohorts or patients with ischemic heart disease, this study enrolled all consecutive clinical CMR referrals. This broader, unselected cohort enhances generalizability and minimizes selection bias. Furthermore, this study extended the evaluation to include LV strain parameters (GRS, GCS, and GLS), enabling a comprehensive and multi-faceted evaluation of the utility and reliability of DL cine. Although there were statistically significant differences in LV strain indices, the effect sizes of GRS, GCS, and GLS were classified as small and within acceptable limits, suggesting negligible impact on diagnostic interpretation. Nonetheless, when using single-BH and FB-MOCO DL cine, the evaluation of strain needs to be treated with caution and should be comprehensively assessed in combination with clinical data and other relevant examinations.

Regarding the grading of LV function impairment based on the estimation of EF, the single-BH DL cine resulted in 3 patients out of 86 was misclassified as better and 3 as worse compared to the reference standard. The FB-MOCO DL cine misclassified 5 patients as having worse EF. These errors stemmed from LVEF differences of 5.8%–6.9%, a range consistent with known operator variability of approximately 3%–7% when measuring EF with conventional multi–breath-hold cine MRI [8], [40]. In other words, roughly 5% of variability in EF measurements can be attributed to normal measurement error, rather than systematic bias in the DL methods [32], [41].

The overall image quality of the DL cine sequences matched that of the segmented reference, while the three-dimensional volumetric mesh scored higher. Conventional segmented cine assembles ventricular images slice-by-slice, then stitches them together. It is a process vulnerable to breath-hold variability, timing mismatches, and the constraints of traditional reconstruction algorithms when confronted with complex cardiac motion. Such factors can introduce gaps or inaccuracies in the resulting 3D meshes, lowering quality score [28]. In contrast, DL cines’s adaptive sampling methods and learned reconstruction seamlessly integrate spatio‐temporal information across the entire cardiac cycles. They can intelligently fill in the missing information and sharpen blurred regions to produce more complete and accurate ventricular mesh.

Nevertheless, it is noteworthy that the DL cine is not without trade-offs. The aggressive undersampling required for high acceleration diminishes sampling frequency, which can blur myocardial borders and reduce blood-myocardium contrast and edge sharpness compared to segmented cine. Lower sampling density might fail to resolve subtle signal variations between the BP and myocardial tissue, compromising BMC calculations. Meanwhile, it also causes the loss of detailed information on the image edges, reducing the edge sharpness [22]. This limitation may introduce inaccuracies in endocardial border delineation during post-processing analysis, particularly in basal segments, thereby necessitating increased manual corrections and prolonging workflow inefficiencies. To address this, our future work will develop an AI-powered enhancement framework for DL-accelerated cine sequences, targeting improved boundary delineation accuracy and optimized BMC to minimize operator-dependent adjustments.

## 6 Limitations

This study has several limitations. First, the study only included patients from a single center and used a single magnetic resonance device. As this is a preliminary clinical application, in the future, it is necessary to include a larger sample size from multiple centers for validation to eliminate potential selection bias. Second, this study did not include pediatric patients who may benefit the most, which will be corrected in subsequent research.

## 7 Conclusion

This study demonstrates that two DL cine sequences can achieve diagnosable IQ, accurate ventricular morphology and function indexes under single-BH or FB conditions, with significantly shortened scanning times without compromising diagnostic performance. These sequences demonstrate potential as rapid cardiac cine alternatives, particularly valuable for patients with compromised respiratory function who struggle with repeated breath-holds or prolonged examinations. They significantly reduce required breath-holds from ∼9 in conventional segmented BH sequences to 0–1.

## Funding

This work was supported by the National Natural Science Foundation of China (82272068 to Yi He).

## Author contributions

Zekun Zhou: Visualization, Investigation. Zhaozhao Wang: Visualization, Investigation. Huihui Kong: Writing – review & editing, Writing – original draft, Data curation. Jianmin Yuan: Supervision. Jinchen Li: Validation, Software. Guang Li: Validation, Software. Dan YU: Supervision. Yi He: Writing – review & editing, Methodology, Conceptualization. Xiangming Li: Visualization, Investigation.

## Ethics approval and consent

Institutional Review Board approval was obtained. The study protocol was approved by the Ethics Committee of Beijing Friendship Hospital (No. BFHHZS20250088).

## Consent for publication

Written informed consent was obtained from all patients in this study. All data and material are available.

## Declaration of competing interests

The authors declare that they have no known competing financial interests or personal relationships that could have appeared to influence the work reported in this paper.
