# Supplementary material for: Clinical feasibility of two cardiac deep learning cine magnetic resonance imaging sequences: Single-breath-hold and free-breathing motion-corrected approaches
Source: J Cardiovasc Magn Reson. 2025 Oct 31;27(2):101983. doi: 10.1016/j.jocmr.2025.101983 (PMC12766484; doi:10.1016/j.jocmr.2025.101983)
Supplement: Supplementary file 1 — Supplementary material [file mmc1.docx]

Supplementary Table. Detailed acquisition time for Three Cine Sequences.

|  | segmented cine | single-BH cine | Percentage reduction (segmented cine vs. single BH cine) | FB-MOCO cine | Percentage reduction(segmented cine vs. FB cine) |
| --- | --- | --- | --- | --- | --- |
| short-axis AT | 90.95 (77.75, 103.13) | 17.76 ± 3.14 | 80% | 83.00 (72.45, 95.78) | 9% |
| long-axis AT | 25.50 (23.03, 27.73) | 5.65 (5.10, 6.13) | 78% | 7.97 (6.90, 9.00) | 69% |
| short-axis total AT | 174.50 (160.10, 192.25) | 17.76±3.14 | 90% | 83.00 (72.45, 95.78) | 52% |
| long-axis total AT | 65.50 (63.03, 67.73) | 45.65 (45.10, 46.13) | 30% | 7.97 (6.90, 9.00) | 91% |
| total AT (short and long axis) | 239.70 (224.55, 260.15) | 63.55 (60.98, 66.00) | **73%** | 90.65 (79.43, 103.80) | **62%** |

AT, acquisition time. Total AT includes BH instructions and rest time between BHs.

**Materials and Methods**

**Data acquisition and image reconstruction of the DL-cine**

**The initial real-time reconstruction.** By “initial real-time reconstruction,” we mean the preliminary reconstruction of the cine images immediately after data acquisition, performed on a frame-by-frame basis in near-real-time. In practice, the undersampled k-space data from the free-breathing scan are first reconstructed into a series of images with full temporal resolution (e.g., one image per heartbeat frame) without any respiratory motion correction. This serves as a first-pass or baseline reconstruction using our network’s real-time mode, producing a raw cine series that still contains respiratory motion artifacts. The sole purpose of this initial reconstruction is to provide images from which we can estimate motion (respiratory displacement) – it is not the final high-quality output. This first stage yields a quick, uncorrected image series used for motion tracking, distinct from the subsequent motion-corrected reconstruction.

**2D non-rigid registration.** The motion correction (MOCO) algorithm used in our study is a non-AI, non-rigid registration algorithm based on a well-established optimization framework. This specific MOCO algorithm is implemented as part of the commercial RegEngine toolset provided by United Imaging Healthcare. The core algorithm itself is based on prior published work. We have now explicitly referenced the foundational paper for this method: Lorenzi M, Ayache N, Frisoni GB, Pennec X; Alzheimer's Disease Neuroimaging Initiative (ADNI). LCC-Demons: a robust and accurate symmetric diffeomorphic registration algorithm. Neuroimage. 2013 Nov 1;81:470-483.

Reference 29. Lorenzi, M., et al., LCC-Demons: a robust and accurate symmetric diffeomorphic registration algorithm. NeuroImage, 2013. 81: p. 470-483.

**Motion-corrected images.** In our workflow, each frame in the initial cine series is deformably registered to a common reference frame. In practice, we choose a reference at a consistent respiratory phase and register all other frames to this reference geometry. This produces a set of deformation fields that warp every frame into alignment with the reference frame. Using these fields, we then warp each original frame to create a motion-corrected version of the frame, now all aligned to the reference respiratory phase. These motion-corrected images are indeed the result of applying the estimated deformations – effectively, the original images have been resampled/warped to compensate for respiratory motion. We emphasize that these motion-corrected images are intermediate inputs to the final reconstruction network rather than final outputs themselves. In the final step of our pipeline, the aligned (motion-corrected) frames are fed into the deep learning reconstruction network which combines the information from all these aligned frames to produce the final high-quality cine series.

**Temporal interpolation**. In our pipeline, after obtaining the deformation fields from the non-rigid registration, we perform linear temporal interpolation along the time axis of the image series. The user can specify the desired number of output phases (frames) per cardiac cycle – in our study we chose a fixed number (for example, 25 phases per heartbeat, matching a typical cine frame rate). As a result, the effective temporal resolution of the final cine series is increased. In our implementation, the interpolation typically doubles the frame count relative to the acquired frames. For instance, if the real-time acquisition provided ~12 frames over the cardiac cycle, we interpolate to ~24 frames (phases), achieving roughly a two-fold finer temporal sampling. In terms of time resolution, our output cine frames have an approximate temporal resolution of ~25–30 ms (depending on heart rate, equals to RR/phases), which is comparable to conventional breath-held cine. We opted for linear interpolation given the short frame-to-frame intervals in our data (on the order of tens of milliseconds), where higher-order spline interpolation did not show significant benefit.

**The training data flow and processing steps.** Briefly, our network was trained in a supervised fashion using retrospectively undersampled breath-hold cine data. The training set consisted of fully sampled short-axis cine MRI from healthy volunteers, acquired with standard segmented 2D cine sequences under breath-hold. These fully sampled images served as the ground truth reference, which refer to conventional high-quality cine MRI, typically obtained over multiple breath-holds to cover all slices. We then retrospectively applied the VALAS undersampling pattern to these data to simulate the undersampled acquisitions, and the network was trained to reconstruct the cine frames from this undersampled input. Importantly, no motion correction or temporal interpolation was applied to the training data, since the training images were from breath-held scans (with no respiratory motion). The motion-correction and interpolation steps are part of our inference pipeline for free-breathing data only, not part of the training pipeline. Regarding temporal regularization: aside from the intrinsic spatio-temporal modeling built into our network (and the integrated spatial-temporal total variation (STTV) regularizer within the network’s unrolled iterations), we did not impose any additional explicit temporal regularization term during training or reconstruction. The network’s loss function and architecture were deemed sufficient to enforce temporal consistency, so no extra smoothing constraints (such as an external temporal TV in the loss) were used.

**The technical details of the applied DC layer.** In our unrolled network, the DC layer is the module that enforces fidelity to the measured k-space data at each iteration. The data-consistency enforcement is integrated into each iteration of our Res-CRNN reconstruction architecture. By doing so, we ensure that the network cannot stray from the true measured data – it can only fill in and refine the missing information. The DC layer is implemented in a manner that is fully differentiable, allowing gradients to propagate through it during training. This property, combined with our network’s residual connections, helps to constrain high-frequency details (such as myocardial edges) to match the measured data and suppresses artifacts. We trust that this added description clarifies the role and implementation of the DC layer, in line with (and elaborating on) the approach in Reference 31.

**Res-CRNN (Residual Convolutional Recurrent Neural Network) framework**. our reconstruction network is built on the same Res-CRNN (Residual Convolutional Recurrent Neural Network) framework as outlined in Reference 31. Additionally, our network includes a spatial-temporal total variation prior and a two-level residual architecture, which are tailored to our cine application. Specific parameters for training: the learning rate is 0.001, multiplied by 0.8 every 80 epochs, and the batch size is 1.

Reference 31. Cheng, J., et al., A dynamic approach for MR T2-weighted pelvic imaging. Phys Med Biol, 2024. 69(20).
